# Viral suppression among adolescents on HIV treatment in the Sedibeng District, Gauteng province

**DOI:** 10.4102/curationis.v45i1.2312

**Published:** 2022-09-27

**Authors:** Sibongile Mabizela, Brian van Wyk

**Affiliations:** 1 School of Public Health, Faculty of Community and Health Sciences, University of the Western Cape, Bellville, South Africa

**Keywords:** HIV, AIDS, adolescents, viral suppression, adherence, antiretroviral therapy

## Abstract

**Background:**

Progress has been made to increase access to antiretroviral therapy (ART) for adolescents living with HIV (ALHIV) to improve their survival, but ALHIV still have worse treatment adherence and viral suppression compared to adults and children.

**Objective:**

To determine the prevalence of viral suppression and the associated factors among adolescents aged 10–19 years on ART at an urban public primary healthcare facility in the Sedibeng district, Gauteng.

**Method:**

A cross-sectional survey was conducted among 192 adolescents who were on ART for at least six months between 2015 and 2018. A self-developed data extraction tool was used to collect data from the Tier.Net electronic database and clinical folders. Data were captured on Microsoft Excel, and descriptive and inferential analyses were performed using SPSS 27 statistical software.

**Results:**

The median age at ART initiation of adolescents was 9.0 years (interquartile range [IQR]: 5.0–12.0), and the median duration on ART was 70.5 (IQR: 30.25–105.5) months. The prevalence of viral suppression (< 1000 copies/mL) among adolescents on ART was 74%, with 41% achieving full suppression (< 50 copies/mL). Those adolescents who reported optimal ART adherence were more likely to be virally suppressed compared to those who reported poor adherence (98.1% vs 25.0%; *p* ≤ 0.001).

**Conclusion:**

Adolescent viral suppression of 74% is higher than in comparable sites, but still way too short of the UNAIDS target of 90%. We recommend adherence support for adolescents to achieve viral suppression.

**Contribution:**

The study highlights the urgent need for targeted adherence support interventions for adolescents living with HIV on antiretroviral therapy to improve rates of viral suppression to meet UNAIDS target of 95%.

## Introduction

An estimated 1.75 million adolescents were living with human immunodeficiency virus (HIV) in 2020 globally (United Nations International Children’s Emergency Fund [UNICEF] 2021), making HIV the most significant public health challenge. Eastern and Southern Africa are the regions most affected with HIV and acquired immune deficiency syndrome (AIDS). Approximately 120 000 (31 000–250 000) adolescents were newly infected with HIV in 2018 and 1.1 million (750 000–1.6 million) adolescents living with HIV (UNICEF 2019). Among the Eastern and Southern African countries, South Africa (SA) had the highest number of children and adolescents (aged 0–19 years) living with HIV in 2018 at an estimated 460 000 (UNICEF 2019).

The introduction of antiretroviral therapy (ART) by the South African Department of Health for people living with HIV has provided HIV-positive adolescents with an opportunity for survival and long-term well-being (Cluver et al. 2016). The purpose of ART is to suppress the replication of the virus, restore the immune function, reduce the risk of onward HIV transmission and prolong the average life expectancy of HIV-infected individuals (Edessa, Sisay & Asefa 2019). Monitoring of patients on ART is crucial in the evaluation of the effectiveness of HIV treatment (Johnson 2012). South Africa performs the initial viral load (VL) test at six months after ART initiation and repeats every 12 months thereafter (Kubheka, Archary & Naidu 2020). Viral monitoring is critical for early detection of treatment failure and to signal for enhanced adherence counselling (EAC) to be implemented (World Health Organization [WHO] 2016).

Viral suppression for children under 15 years ranged from 40% to 90% in low- and middle-income countries (LMICs) (Kadima et al. 2019). In SA, a viral suppression rate of 81% was reported among adolescents and young people aged 15–24 years (Zanoni et al. 2016). Determinants are defined as factors that influence the health of individuals (WHO 2017). In this study, sociodemographic (gender, age, education and social support), clinical (duration on ART, WHO stage, regimen at ART start, baseline and current CD4 counts, history of tuberculosis) and behavioural factors (adherence, alcohol and substance use and HIV disclosure) were analysed as potential factors (determinants) that influence viral suppression among adolescents on ART.

Older adolescents (15–19 years) were reported to have lower rates of viral suppression than younger adolescents (10–14 years) as a result of challenges experienced when transitioning from the paediatric to adult HIV programmes (Van Wyk, Kriel & Mukumbang 2020a). Female patients were more likely to have higher viral suppression than male patients, because men tend to engage in high-risk behaviours such as not using condoms and having multiple sex partners, and men seemed to have poor health-seeking behaviours compared to women (Fokam et al. 2019).

Adolescents whose parents were still alive had better viral suppression due to positive parental support (Dixon-Umo & Ikpeme 2020). These adolescents were reminded by parents to take their antiretroviral (ARV) drugs or refill ARV prescriptions, and sometimes parents accompanied adolescents to the clinic for appointments (Knodel et al. 2010).

Low viral suppression was common in patients with weakened immune systems, who present with opportunistic infections, on WHO stages III or IV and/or a low CD4 count (< 200 cells/µL) upon initiation on ART. In South Africa, patients on tuberculosis (TB) treatment were reported to have very low viral suppression (Joseph Davey et al. 2018), while in Kenya, TB co-infection was found to be associated with better virologic outcomes (Kadima et al. 2019), which may be due to directly observed therapy and adherence support.

Since 2010, the South African guidelines replaced stavudine (d4T) with abacavir (ABC) for children and young adolescents and d4T with tenofovir (TDF) for older adolescents due to concerns around its toxicity (National Department of Health [NDOH] 2015; Technau et al. 2013). The guidelines recommended efavirenz (EFV)-based regimens for children below 15 years as they were more likely to achieve viral suppression compared to being on nevirapine (NVP) – or lopinavir or ritonavir LPV/r-based regimens. In Tanzania and Kenya, protease inhibitor (PI)-based regimens were associated with viral suppression and prevention of drug resistance mutation (DRM) compared to non-nucleoside reverse transcriptase inhibitor (NNRTI)-based regimens (Kadima et al. 2019; Muri et al. 2017), suggesting that PI-based regimens have good performance in viral suppression.

High levels of adherence to therapy are essential to achieve and sustain viral suppression and prevent disease progression to AIDS (Ghanbari et al. 2019). Poor adherence as a result of poor palatability of drugs, the lack of clinicians’ confidence in treating children with HIV and inadequate psychological support represent the major reasons for failure to follow up and virological failure (VF) in children and adolescents (Fokam et al. 2019; Kadima et al. 2019). Retention in care (RiC) is a critical precursor to viral suppression, as it helps maintain adherence, while failure to be retained or interruptions to ART increase the risk of drug resistance and mortality (Murray et al. 2017).

### Problem statement

Despite the successful rollout of ART in SA, adolescents on HIV treatment demonstrate poor ART adherence, retention in care (RiC) and viral suppression as compared to adults and children (Van Wyk & Davids 2019; Van Wyk et al. 2020a). Human immunodeficiency virus treatment programmes are designed for children under 15 years or adults and do not cater for the specific needs of adolescents (Van Wyk, Kriel & Mukumbang 2020b). Additionally, routine monitoring of HIV treatment does not report treatment outcomes for adolescents (aged 10–19 years), as reporting is only for children under 15 years or adults (15 years and older). It is thus difficult to assess the performance of adolescents in the HIV treatment programme. Specific analysis is required to combine information for adolescents from the paediatric and adult HIV treatment programmes, to inform proper planning for adolescent-specific interventions and to identify factors that influence treatment outcomes for adolescents on ART in public healthcare settings in South Africa. At the time of the current study, no such study has been conducted in the Sedibeng district.

### Objective of the study

The objective of the study was to determine the prevalence of viral suppression and the factors associated with viral suppression among adolescents aged 10–19 years old on ART at an urban public primary health care facility in Sedibeng district, Gauteng.

### Research setting

The study was contextual, as it was conducted at a specific healthcare facility in Sedibeng district. Sedibeng district is located in the southern part of the Gauteng province and consists of three local municipalities: Emfuleni, Midvaal and Lesedi. Among these, the Emfuleni municipality has the highest population of more than 700 000 people (eds. Massyn et al. 2017; Sedibeng District Municipality n.d.). Many people live in the township areas, especially Sebokeng and Evaton. Levai Mbatha Community Health Centre (CHC) is a referral healthcare facility located in Evaton township and provides comprehensive healthcare services to over 220 000 residents, of whom more than 80% are uninsured and depend on free public primary health care services (Akinsanyaa et al. 2017; Phukuta & Omole 2020).

## Research method and design

### Design

A retrospective cross-sectional study was conducted using routine records of adolescents who were on ART at an urban public primary health care facility in Gauteng between January 2015 and December 2018. The cross-sectional study permitted selecting participants based on the inclusion and exclusion criteria set and aided in determining the prevalence of adolescent viral suppression.

### Population and sampling

Routine records of adolescents (10–19 years) who were on ART for 6 months or more at the public primary health care facility in Gauteng and contained at least one HIV viral load result were included in the study. The latest viral load result recorded between January 2015 and December 2018 was considered. Records of adolescents who had died, were lost to follow-up or were transferred to another healthcare facility within six months of ART initiation were excluded from the study.

Using ClinCalc (Kane 2019), a calculator used to determine the minimum number of subjects that need to be enrolled in a study, the minimum sample size of adolescents’ records required was calculated as **186**, with parameters of estimated viral suppression prevalence of 65.5%, predicted viral suppression rate of 75.0%, alpha of 0.05 and power of 80.0%. However, all available records of adolescents on treatment for the period that met the inclusion criteria were included in the analysis.

### Data collection method

A data extraction tool was developed to collect data in two stages: from Tier.Net and from the patient folders and clinic cards. The data extraction tool was developed by the first author, in consultation with the HIV, AIDS, sexually transmitted infections (STIs) and TB (HAST) clinician and the study supervisor. The HAST clinician advised on which variables from Tier.net provide valid and reliable measures. The study supervisor advised on available individual patient information based on his experience of conducting similar studies in the Western Cape, Free State and Mpumalanga.

In the first stage, clinical data was extracted from the Tier.Net electronic system. The Tier.Net is a three-tiered nation-wide health information system comprising of paper-based, standalone electronic and networked electronic medical record systems, which was developed for routine monitoring and evaluation of public HIV treatment programmes in South Africa (Myburgh et al. 2015). Each tier produces the same nationally required monthly enrolment and quarterly cohort reports so that outputs from the three tiers can be aggregated into a single database at any level of the health system. The system provides programme managers with an understanding of the burden of care, quality of service, retention in care and other outcomes of the programme (Osler et al. 2014). The district information officer ran the queries on Tier.Net based on the specifications for the study (eligibility criteria for participants). Variables obtained from Tier.Net were gender, duration on ART, WHO stages, regimen at ART start, baseline and current CD4 counts, and viral load results. Outputs from Tier.Net were transcribed into an Excel spreadsheet. To ensure reliability of the data, the first author double-checked the spreadsheets for transcription errors.

The second stage of data collection consisted of a review of patient folders and cards to extract sociodemographic (education, age and social support), clinical (history of TB) and behavioural-related (adherence, disclosure and alcohol and substance use) information.

### Data analysis

Descriptive and inferential analyses were performed using Statistical Package for Social Sciences (SPSS) version 27 (IBM Corporation) statistical software package and the prevalence of viral suppression was determined by using Microsoft Office Excel. The primary outcome was dichotomised into viral suppression (< 1000 copies/mL) and viral nonsuppression (≥ 1000 copies/mL). Characteristics of the study population were described using frequency tables. Before running the frequency analyses, data were coded, and where a variable contained missing values, these missing values were coded as discrete missing values using ‘999’ so that SPSS could recognise them as completely missing.

Continuous variables such as age, CD4 count and duration on ART were also summarised together with their median and interquartile ranges (IQR). The chi-square or Fischer’s exact tests for categorical variables and Mann–Whitney test for continuous variables were performed to determine the statistically significant relationship between independent and dependent variables. Variables with a significance of ≤ 0.05 were considered associated with viral suppression.

### Ethical considerations

The University of the Western Cape Biomedical Research Ethics Committee approved the study protocol (reference number BM18/5/14). Permission to conduct the study at the public primary health care facility was granted by the Sedibeng District Health Research Committee.

Informed consent was not required from the patients, as there was no direct contact with them.

Anonymity was ensured by not using patients’ names and folder numbers, and a coding system was used when data were analysed and reported. Confidentiality was maintained throughout the study, and only the researcher had access to the databases.

## Results

### Realisation of sample

**FIGURE 1:**
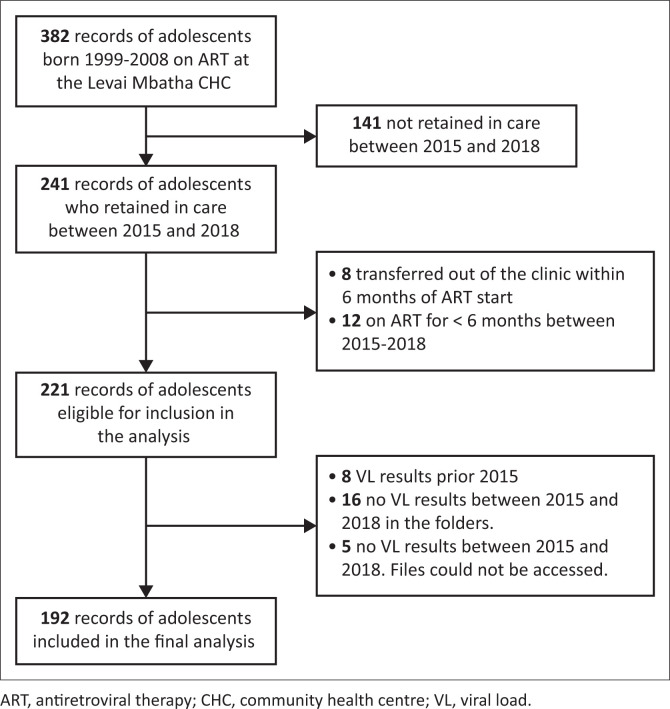
Selection of adolescents into the study.

Records of 382 adolescents attending ART services at the primary health care facility were obtained from the Tier.Net. Figure 1 shows that of 382 records, 241 records were of adolescents who were in care during the period 2015–2018, while 141 records were of adolescents who were not in care during 2015–2018. Of the 241 records, eight records were of adolescents who transferred out of the clinic within 6 months of ART initiation, and 12 records were of adolescents who were on ART for less than six months between 2015 and 2018. Records of 221 adolescents were eligible for inclusion in the study; however, eight records were of adolescents who had their last VL results available before 2015; 16 records were of adolescents who had no VL results between 2015 and 2018 captured on Tier.Net and in their clinical folders; and five records were of adolescents who had no VL results between 2015 and 2018 captured on Tier.Net, and the patients’ folders could not be found to track the results for the period of study. Records of 192 adolescents were included in the final analysis.

### Sociodemographic and clinical characteristics of adolescents on antiretroviral therapy

Table 1 shows the sociodemographic and clinical characteristics of adolescents. The median age of adolescents on ART was 15.0 years (interquartile range [IQR]: 12.25 –17.0). Over half of the adolescents were 15–19 years (*n* = 103; 53.6%) and female (*n* = 105; 54.7%). The median age at ART initiation of adolescents was 9.0 years (IQR: 5.0–12.0), with 109 (57.0%) adolescents initiated on ART between 0 and 9 years. Education attainment was only recorded for 11 adolescents, with nine having primary or secondary education recorded and two not attending any school.

**TABLE 1:** Sociodemographic, clinical and behavioural characteristics of adolescents on antiretroviral therapy at a public primary health care facility in Sedibeng district, Gauteng (*n* = 192).

Characteristics	Frequency	%
**Age (in years)**		
10–14	89	46.4
15–19	103	53.6
**Gender**		
Male	87	45.3
Female	105	54.7
**Education**		
Primary or secondary	9	4.7
No education	2	1.0
Missing	181	94.3
**Support**		
Family	144	75.0
Other	3	1.6
Missing	45	23.4
**WHO stage at ART initiation**		
Stage I or II	74	38.5
Stage III or IV	83	43.2
Unknown	35	18.2
**Previous history of TB**		
Yes	28	14.6
No	108	56.3
Missing	56	29.1
**Baseline CD4 count (cells/µL)**		
< 350	87	45.3
≥ 350	46	24.0
Missing	59	30.7
**Current CD4 count (cells/µL)**		
< 350	40	20.8
≥ 350	146	76.1
Missing	6	3.1
**TB treatment at ART start**		
Yes	7	3.6
No	112	58.4
Missing	73	38.0
**Other medication**		
Yes	4	2.1
No	6	3.1
Missing	182	94.8
**Duration on ART (in months)**		
≤ 12	17	8.9
13–24	23	11.9
≥ 25	152	79.2
**Regimen at ART start**		
NNRTI-based	179	93.2
PI-based	13	6.8
**HIV disclosure**		
Yes	144	75.0
No	2	1.0
Missing	46	24.0
**Adherence**		
Good	105	54.7
Poor	52	27.0
Missing	35	18.2
**Alcohol use**		
Yes	1	0.5
No	29	15.1
Missing	162	84.4
**Substance use**		
Yes	1	0.5
No	24	12.5
Missing	167	87.0

ART, antiretroviral therapy; TB, tuberculosis; CD4, cluster of differentiation 4; NNRTI, non-nucleoside reverse transcriptase inhibitor; PI, protease inhibitor; HIV, human immunodeficiency virus.

The majority (*n* = 144; 75.0%) of adolescents were supported by family with regard to treatment collection and clinic visits, while three (1.6%) adolescents were supported by other people outside their household.

Of the adolescents (*n* = 157, 81.8%) who had WHO staging recorded at ART initiation, 83 (43.2%) presented with stage III and IV, and 74 (38.5%) presented with stage I and II. Only 28 (4.6%) adolescents were recorded to have a history of active TB.

Baseline (at ART initiation) CD4 count was not recorded for 59 (30.7%) adolescents. Of those with recorded CD4 count, 87 (45.3%) were initiated with a count of less than 350 cells/µL.

The majority (*n* = 146; 76.1%) of adolescents had the latest CD4 count greater than 350 cells/µL.

At ART initiation, only seven (3.6%) adolescents were recorded to be on TB treatment. Of the adolescents who had other medication recorded, four (2.1%) were taking other medication concurrently with ART.

The median duration on ART was 70.5 (IQR: 30.25–105.5) months, and most adolescents (*n* = 152; 79.2%) were on ART for longer than 25 months. The majority (*n* = 179; 93.2%) of adolescents were initiated on NNRTI-based regimen, with 48 (64.0%) adolescents on *Abacavir, Lamivudine and Efavirenz* regimen at ages 10–14 years and 33 (89.0%) adolescents initiated on fixed-dose combination regimen *tenofovir, emtricitabine or lamivudine and efavirenz* at ages 15–19 years. Thirteen (6.8%) adolescents were initiated on a PI-based regimen.

Most (*n* = 144; 75.0%) adolescents with recorded HIV status disclosure had disclosed to someone, with 139 (97.0%) disclosing to a family member. Seventy-five (52.0%) adolescents with HIV status disclosed were in the age group 10–14 years.

Over half (*n* = 105; 54.7%) of adolescents were recorded to have good adherence to ART over a period of time, with 60 (57.0%) female adolescents reporting good adherence compared to male adolescents (*n* = 45; 43.0%).

Alcohol and substance use were poorly recorded, with only one (0.5%) reporting alcohol use and one (0.5%) using illegal substances.

### Characteristics of adolescents with viral suppression

The proportion of adolescents with viral suppression was 74% (142), and 41% (58) achieved full suppression at VL < 50 copies/mL at the time of the study. Table 2 illustrates that only adherence had a significant positive association with viral suppression (*p* ≤ 0.001). Viral suppression rates were most favourable among adolescents with good adherence to ART. All other variables were not associated with viral suppression.

**TABLE 2:** Determinants of viral suppression for adolescents on antiretroviral therapy in a primary health care facility in Sedibeng district, Gauteng.

Characteristics	Viral suppression				*p*
	Yes		No		
	*n*	%	*n*	%	
**Age (in years)**					0.207
10–14	62	69.7	27	30.3	
15–19	80	77.7	23	22.3	
**Gender**					0.151
Female	82	78.1	23	21.9	
Male	60	69.0	27	31.0	
**Education**					1.000**
Primary or secondary	7	77.8	2	22.2	
No education	2	100.0	0	0.0	
**Support**					0.560**
Family	103	71.5	41	28.5	
Other	3	100.0	0	0.0	
**WHO stage at ART initiation**					0.435
Stage I or II	54	73.0	20	27.0	
Stage III or IV	65	78.3	18	21.7	
**History of TB**					0.605
Yes	19	70.4	8	29.6	
No	82	75.2	27	24.8	
**Baseline CD4 count (cells/µL)**					0.249
< 350	62	71.3	25	28.7	
≥ 350	37	80.4	9	19.6	
**Current CD4 count (cells/µL)**					0.071
< 350	25	62.5	15	37.5	
≥ 350	112	76.7	34	23.3	
**TB treatment at ART initiation**					0.197**
Yes	7	100.0	0	0.0	
No	84	75.0	28	25.0	
**Other medication**					0.467*
Yes	4	100.0	0	0.0	
No	4	66.7	2	33.3	
**Duration on ART (months)**					
≤ 12	14	82.4	3	17.6	0.455**
13–24	19	82.6	4	17.4	
≥ 25	109	71.7	43	28.3	
**Regimen at ART start**					0.520**
NNRTI-based	131	73.2	48	26.8	
PI-based	11	84.6	2	15.4	
**HIV disclosure**					0.568**
Yes	105	73.4	38	26.6	
No	3	100.0	0	0.0	
**Adherence**					< 0.001*
Good	103	98.1	2	1.9	
Poor	13	25.0	39	75.0	
**Alcohol use**					1.000**
Yes	1	100.0	0	0.0	
No	25	86.2	4	13.8	
**Substance use**					1.000**
Yes	1	100.0	0	0.0	
No	22	91.7	2	8.3	

WHO, World Health Organization; ART, antiretroviral therapy; TB, tuberculosis; CD4, cluster of differentiation 4; NNRTI, non-nucleoside reverse transcriptase inhibitor; PI, protease inhibitor; HIV, human immunodeficiency virus.

* , Indicates statistical significance.

** , Indicates Fisher’s exact test.

## Discussion

This study sought to determine the prevalence of viral suppression and the factors associated with viral suppression among adolescents aged 10–19 years old on ART at the Levai Mbatha Community Health Centre (CHC). The viral suppression rate of adolescents on ART at the Levai Mbatha CHC was 74.0%, which fell short of the UNAIDS target of 90%. Similar results were reported in Ehlanzeni district, in Mpumalanga province, South Africa (Okonji et al. 2021), with a viral suppression rate of 74.3%. However, the suppression rate of this study was higher compared to that in Uganda at 65.5% (Natukunda et al. 2019).

More female than male adolescents were reported in this study, which reflects the higher HIV prevalence among adolescent girls and young women globally (UNAIDS 2016). However, there was no association between gender and viral suppression, even though other studies found an association, possibly due to gender inequalities experienced by adolescents seeking care.

More older adolescents (15–19 years) were on ART and achieved higher viral suppression rates compared to younger adolescents (10–14 years). The high number of older adolescents in this study may be influenced by the aging of perinatally infected infants who entered HIV care in childhood and an increasing number of older adolescents seeking care who were probably infected through sexual transmission (Maskew et al. 2019). Additionally, the high viral suppression rate among adolescents aged 15–19 years could be because they are able to take their ART medication regularly without supervision and possess self-competence on ART adherence (Okonji et al. 2021).

The main support structure for adolescents in this study included parents or family members. According to Natukunda et al. (2019), studies found that family support improved ART adherence and viral outcomes in LMICs. This could be due to positive parenting approaches (praise and support) and parental supervision of adolescent activities (Cluver et al. 2016).

More adolescents in this study were either moderately or severely symptomatic (WHO clinical stage III–IV) at ART initiation, and many had baseline CD4 counts of < 350 cells/µL. Studies have reported that individuals with low baseline CD4 count and WHO stages III or IV at ART initiation could have delayed HIV diagnosis, which resulted in advanced immune compromise and an increased risk of opportunistic infections compared to individuals with higher baseline CD4 counts and WHO stages I or II. Furthermore, the likelihood of treatment failure was reported to be higher among individuals who were initiated on ART after developing AIDS-related illnesses (WHO stage III or IV) (Chimbetete, Tshimanga & Wellington 2011).

A history of TB at baseline is considered a marker for poor health status, and it identifies patients who will benefit from ART (Komati et al. 2010). In this study, there was no association between viral suppression and a history of TB. However, in Kenya, a history of TB was associated with viral suppression among children under 15 years (Kadima et al. 2019), and Zanoni et al. (2017) associated history of TB with higher retention and viral suppression rates among adolescents attending the adolescent clinic. This could be due to close monitoring and adherence support given for TB patients.

A high number of adolescents in this study were initiated on an NNRTI-based regimen. However, the few adolescents initiated on a PI-based regimen had better viral suppression rates. Muri et al. (2017) reported that a PI-based regimen had good performance in viral suppression and prevention of DRM compared to a NNRTI-based regimen. In contrast, Fokam et al. (2019) reported that patients on TDF+3TC+EFV had the highest virological success compared to those on other first-line (including those that are NVP-based and those containing zidovudine (AZT) and ABC as NRTI-backbone) or PI-based regimens, because EFV-based regimen had excellent virologic efficacy (The Panel on Antiretroviral Guidelines for Adults and Adolescents [PAGAA] 2021). The use of the most suitable regimen for adolescents remains crucial given the risk of poor adherence and treatment failure and the development of drug resistance among this group.

This study found that more adolescents were on ART for longer than 25 months; nonetheless, higher viral suppression was achieved by adolescents on ART for 13–24 months. Different studies have reported variations around the duration on ART with viral suppression; however, it is expected that longer duration on ART among adolescents was associated with viral suppression (< 1000 copies/mL) and full viral suppression (< 50 copies/mL) Haghighat et al. 2021).

The use of other medication alongside ART in this study was not associated with viral suppression. However, Sithole et al. (2018) associated other medications with virological failure. This is explained by the pill burden that would be encountered by adolescents taking other medications with ART.

Many adolescents’ HIV status was disclosed to other people; however, disclosure in this study was not associated with viral suppression. Similarly, Yiltok et al. (2020) found no association in their study, which implied that disclosure was not enough to lead to viral suppression but required the patient to have adequate adherence to ART.

Fewer adolescents in this study were found to be consuming alcohol and/or using substances. No association was found between alcohol and/or substance use and viral suppression in this study; however, other studies have demonstrated that alcohol and/or substance use were associated with the loss of durable viral suppression, greater time spent with a VL >1500 copies/mL and low RiC (PAGAA 2021).

Viral suppression requires effective ART, which implies that the correct dose of appropriate ART is taken consistently and at the correct time interval (Yiltok et al. 2020). In the current study, 98.1% of adolescents with good adherence achieved viral suppression. Studies have established 95.0% adherence levels by pill counts only among adolescents (Nabukeera–Barungi et al. 2015). In this study, adherence may have been higher because clinical records were used to assess adherence. Adherence assessment was initially made by the clinicians during their routine clinical practice. Most ART-servicing facilities are always occupied, and clinicians may not be in a position to precisely assess adherence by counting pill balances from all patients. Studies have shown that the pill count method of adherence assessment can be manipulated, and adherence has been found to differ with the method of assessment used (Nabukeera–Barungi et al. 2015).

## Limitations of the study

The use of a retrospective study design meant that the study could not allow causality to be established. The sample size was too constricted, which limited the ability to conduct a multivariate logistic regression and could have potentially influenced the findings.

The major limitation of this study was missing data, which could have resulted in missing data bias. Missing data from patient clinical folders resulted from incorrect documentation, incomplete folders, duplicate folders and missing clinical folders.

## Recommendations

Further studies need to be conducted to measure adolescents’ progress towards achieving the UNAIDS goals.

The HIV programme needs to carry out monitoring and evaluation (M&E) specifically for adolescents and develop adolescent-specific indicators and outcome measures which would assist in identifying the challenges to reaching the goal of viral suppression. The HIV programme should also consider health literacy on the benefits of ART, especially for asymptomatic adolescents, as it is associated with better adherence to ART. Patients having difficulties with adhering to appointments or ART should be approached in a positive, nonjudgemental and problem-solving manner, and directly observed therapy may also be considered. Regular alcohol and substance screening should be integrated into routine clinical care of all adolescents on HIV treatment. The study recommends greater vigilance in record-keeping by clinicians to ensure accurate and complete recording of patients’ information in patient records. The study also recommends that administrative clerks be held accountable to the National Guideline for Filing, Archiving and Disposal of Patient Records in Primary Health Care Facilities to ensure proper filling of patient folders and avoid having missing or misplaced folders.

## Conclusion

Viral suppression among adolescents on ART was relatively high in this study in comparison to some regions, but it was still below the target of 90%. Adherence was the only factor associated with viral suppression, with more than 95% of adolescents having good adherence. The results highlighted the importance of good ART adherence in improving the viral suppression rate, regardless of the age, gender, duration of ART and ART regimen the adolescent is on. Adherence is particularly critical for viral suppression, as over the long term it can be challenging even for the most motivated adolescents.
